# Supplementary material for: Insights into the operational stability of wide-bandgap perovskite and tandem solar cells under rapid thermal cycling
Source: Nat Commun. 2026 Jan 14;17:596. doi: 10.1038/s41467-025-68219-w (PMC12808105; doi:10.1038/s41467-025-68219-w)
Supplement: Supplementary file 1 — Supplementary Information [file 41467_2025_68219_MOESM1_ESM.pdf]

Supplementary Information

Insights into the Operational Stability of Wide-Bandgap Perovskite and Tandem Solar Cells under Rapid Thermal Cycling

Kun Sun[1,2], Renjun Guo[3,4,], Qilin Zhou[5,6], Lingyi Fang[3,4], Xiongzhuo Jiang[1], Simon A. Wegener,[1] Yuxin Liang[1], Zerui Li[1], Suzhe Liang[7,8], Matthias Schwartzkopf[9], Erkan Aydin[10], Sarathlal Koyiloth Vayali[9,11], Stephan V. Roth[9,12], Ulrich W. Paetzold[3,4], and Peter Müller-Buschbaum[1,*]*

[1]Technical University of Munich, TUM School of Natural Sciences, Department of Physics, Chair for Functional Materials, James-Franck-Str. 1, 85748 Garching, Germany

[2]Present address: Helmholtz-Zentrum Berlin für Materialien und Energie GmbH, Hahn-Meitner-Platz 1, 14109 Berlin, Germany

[3]Karlsruhe Institute of Technology (KIT), Institute of Microstructure Technology, Herrmann-von-Helmholtz-Platz 1, 76344 Karlsruhe, Germany

[4]Karlsruhe Institute of Technology (KIT), Light Technology Institute, Engesserstr. 13, 76131 Karlsruhe, Germany

[5]National University of Singapore, Department of Chemical and Biomolecular Engineering, Singapore, Singapore

[6]National University of Singapore, Solar Energy Research Institute of Singapore (SERIS), Singapore, Singapore

[7]Eastern Institute for Advanced Study, Ningbo Institute of Digital Twin, Eastern Institute of Technology, Ningbo 315200, China

[8]Zhejiang Key Laboratory of All-Solid-State Battery, Ningbo Key Laboratory of All-Solid-State Battery, Ningbo 315200, China

[9]Deutsches Elektronen-Synchrotron, Notkestr. 85, 22607 Hamburg, Germany

[10]Ludwig-Maximilians-Universität München (LMU), Department of Chemistry, Butenandtstr. 11, 81377 München, Germany

[11]Applied Sciences Cluster, University of Petroleum and Energy Studies UPES, Dehradun, Uttarakhand, 248007, India

[12]Royal Institute of Technology KTH, Department of Fibre and Polymer Technology, Teknikringen 56-58, Stockholm, 11428 Sweden

*Corresponding authors: renjun.guo@kit.edu (R.G.); muellerb@ph.tum.de (P.M.B.)

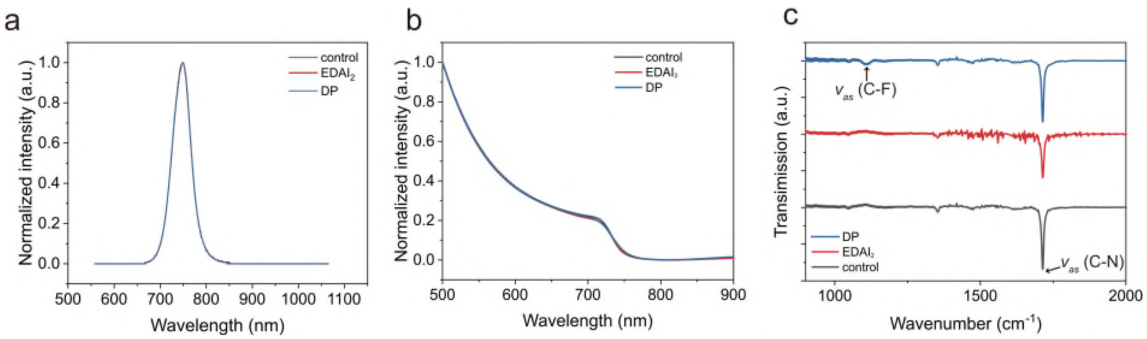

Supplementary Figure. 1. a) Normalized photoluminescence spectra of respective perovskite thin film deposited on glass substrates. b) Absorption spectra of perovskite thin films deposited on glass substrates. c) FTIR spectra of respective perovskite thin films deposited on silicon wafers.

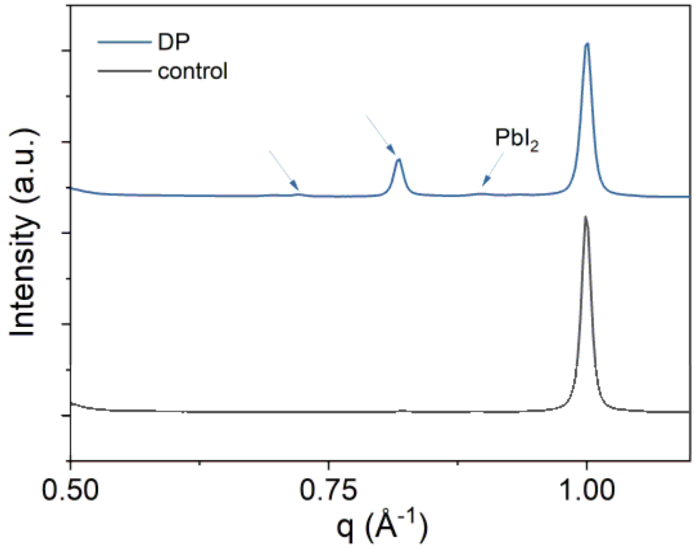

Supplementary Figure. 2. Pseudo XRD data of control and DP devices, with arrows marking the 2D phase formed after dual passivation.

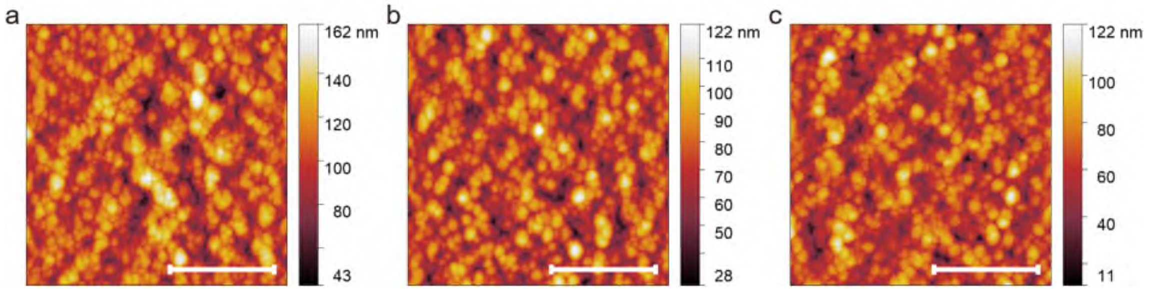

Supplementary Figure. 3. AFM images of a) control perovskite thin film, b) perovskite thin film passivated by $EDAI_2$, and c) perovskite thin films with dual passivation (scale bar is 2μm).

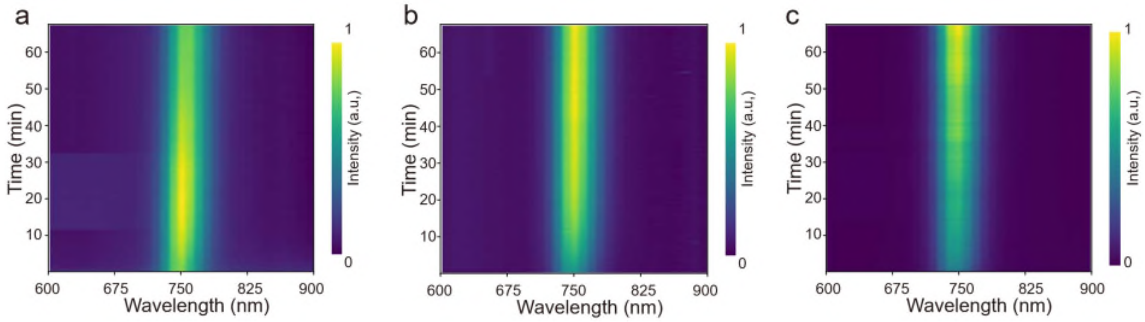

Supplementary Figure. 4. *In situ* time-dependent PL evolution of a) control, b) $EDAI_2$, and c) DP thin films.

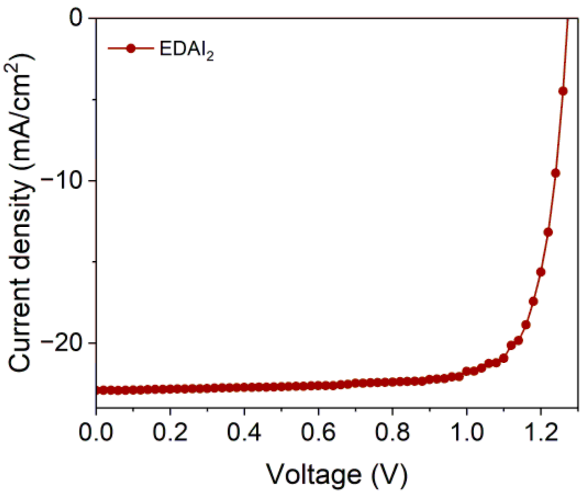

Supplementary Figure. 5. Champion *JV* curve of $EDAI_2$ device under AM 1.5G illumination (100 mW cm^{-2}), retrieved from the reverse scan.

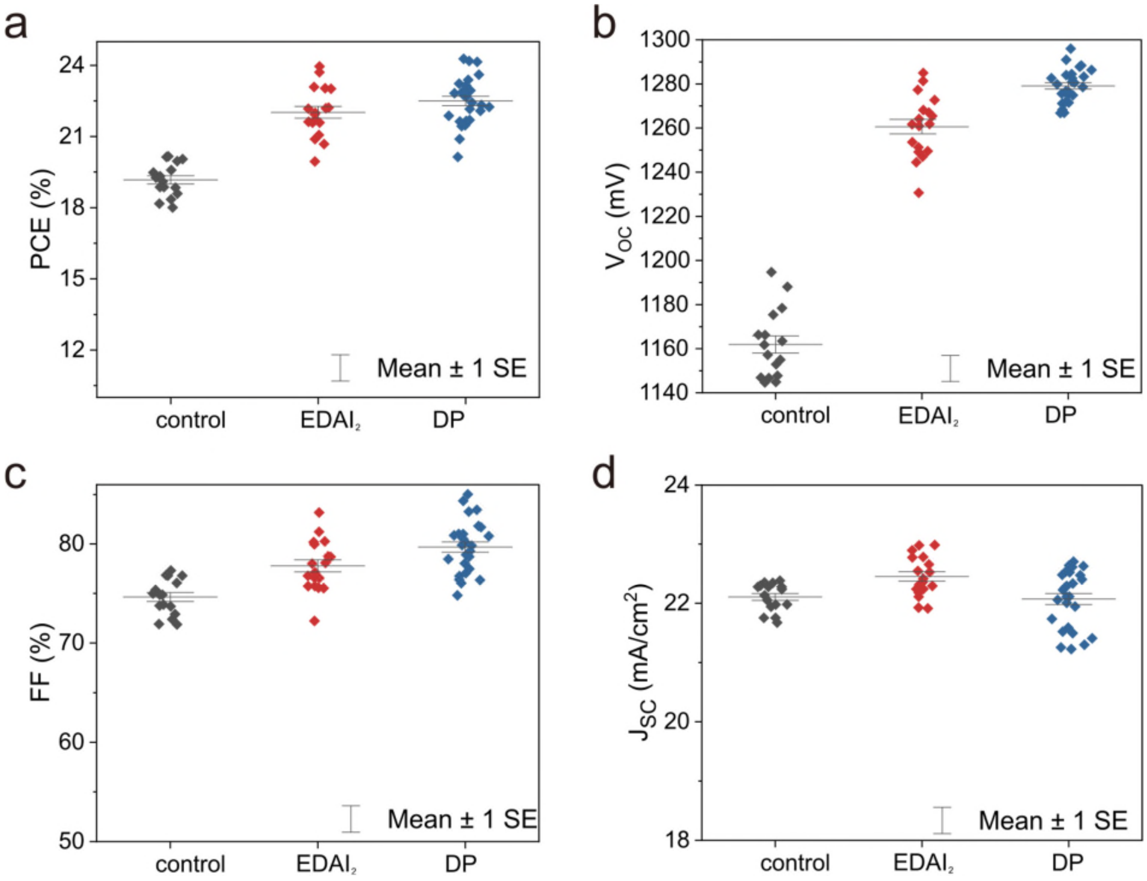

Supplementary Figure. 6. Statistical distribution of device parameters of respective perovskite solar cells: a) PCE, b) V_{OC}, c) *FF*, and d) J_{SC}.

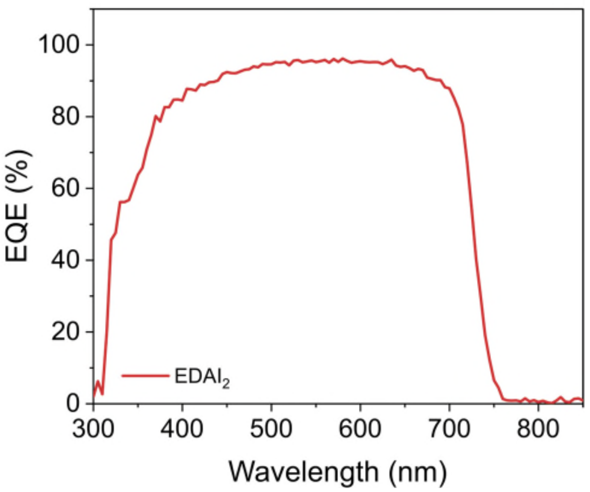

Supplementary Figure. 7. EQE spectra of the champion $EDAI_2$ device.

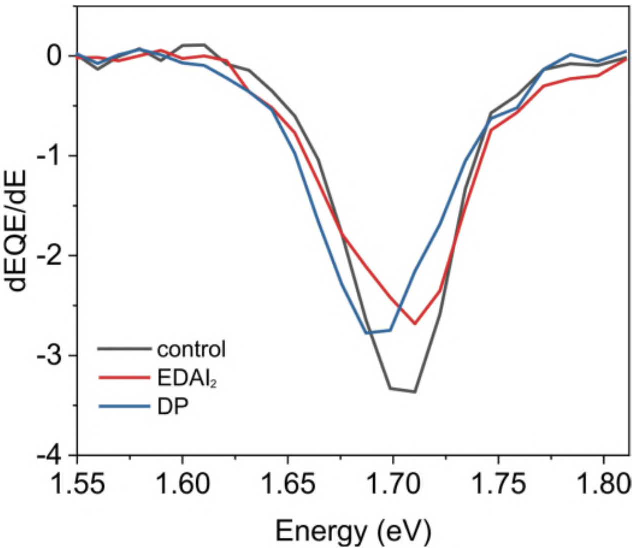

Supplementary Figure. 8. EQE band edge retrieved from the EQE spectra of the respective devices.

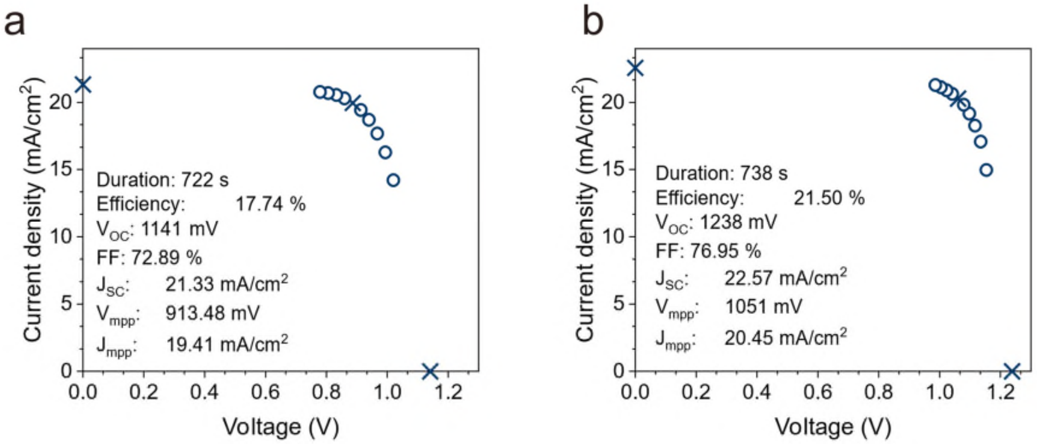

Supplementary Figure.9. Quasi-steady–state measurement of the champion device of a) control and b) $EDAI_2$ type.

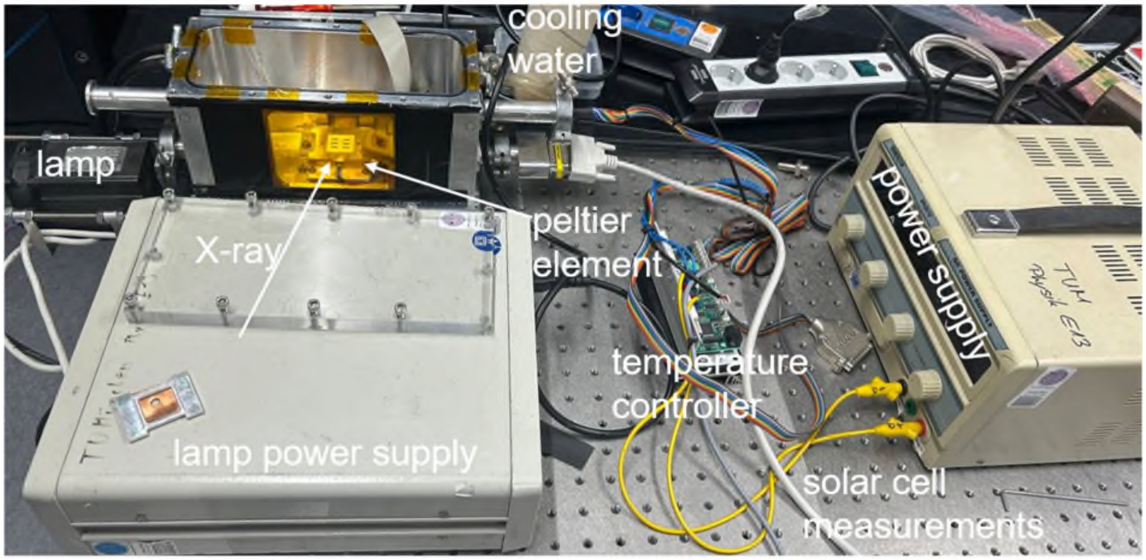

Supplementary Figure. 10. Experimental setup used for monitoring temperature-dependent *J-V* measurements as well as *operando* GIWAXS at DESY, P03. Detailed description can be found in the Characterization section.

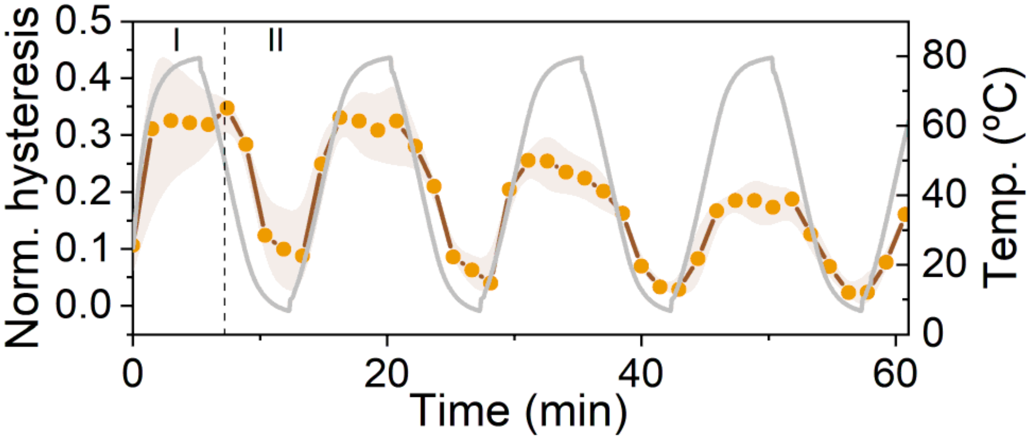

Supplementary Figure. 11. Normalized hysteresis index of DP device and temperature *versus* time. The dashed line divides the process into two regimes: I, initial burn-in, and II, steady degradation.

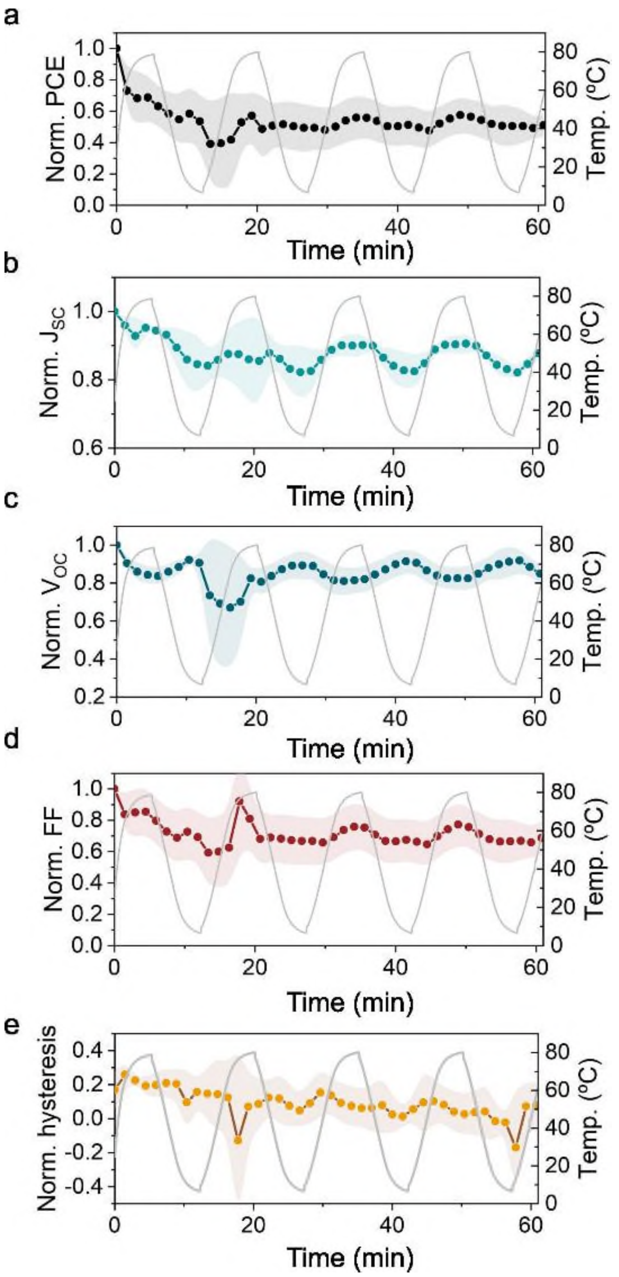

Supplementary Figure. 12. Normalized photovoltaic parameters of control device as a function of time and temperature, where the shaded areas refer to error bars derived from the standard deviation of respective photovoltaic parameters of three pixels subjected to rapid thermal cycling. a) Normalized *PCE* and temperature *versus* time, b) normalized J_{SC} and temperature *versus* time, c) normalized V_{OC} and temperature *versus* time, d) normalized *FF* and temperature *versus* time, and e) normalized hysteresis index and temperature *versus* time.

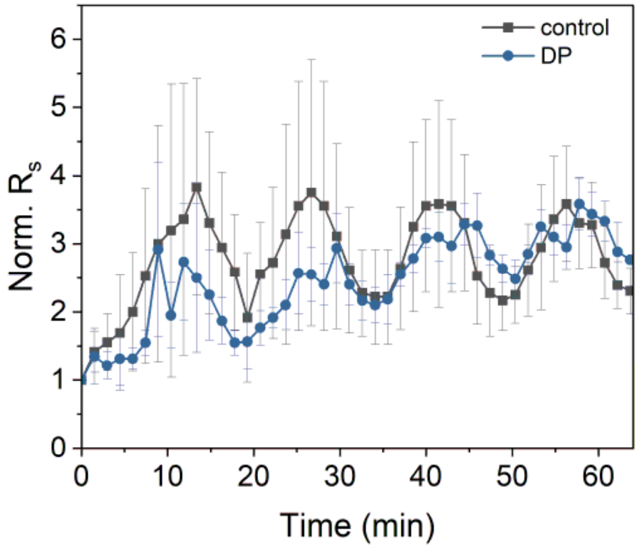

Supplementary Figure. 13. Normalized series resistance of control and DP device as a function of time.

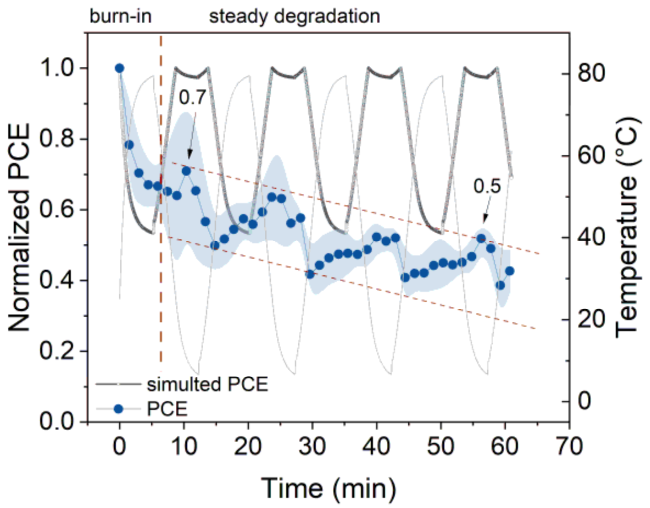

Supplementary Figure. 14. Normalized PCE (5 pixels) and simulated PCE obtained using the nonmonotonic thermal coefficients as a function of temperature and time, where the overall degradation process can be divided into two phases: an initial burn-in phase (30% PCE loss) and a steady degradation phase (20% PCE loss, extracted from the two points indicated by the arrows). The simulation, carried out to model the expected behavior under the experimental temperature profile by assuming no concurrent performance loss, was built using the initial PCE recorded at room temperature and the extracted temperature coefficients for the specified ranges (5−25 °C and 25−85 °C), which were then applied across the measured temperature profile.

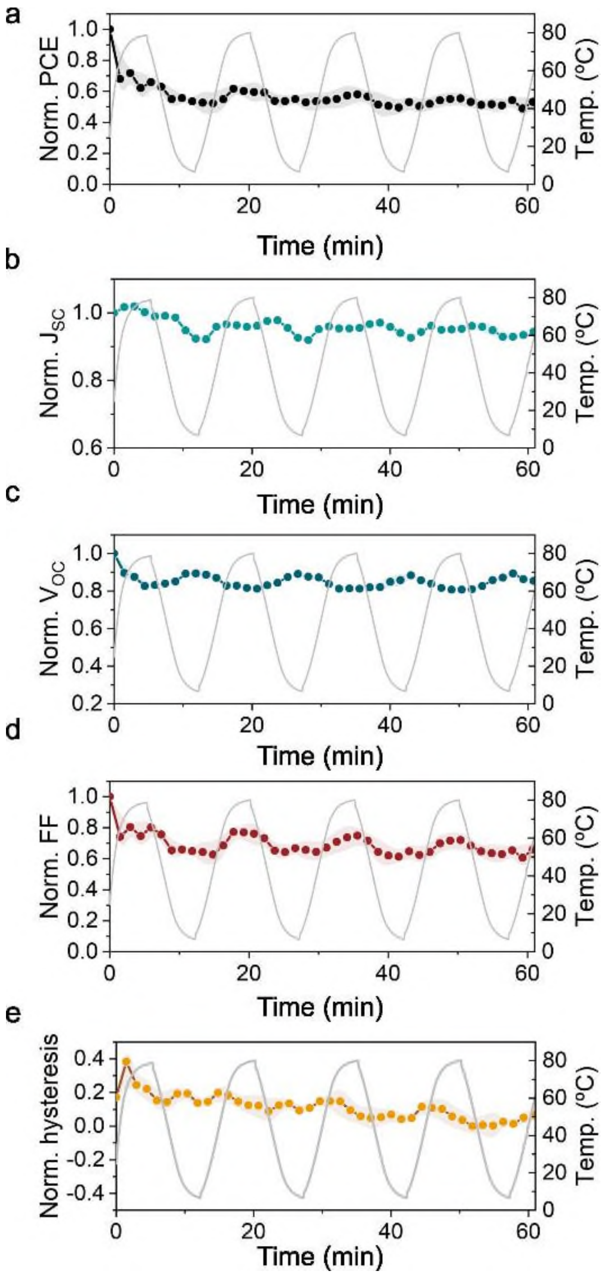

Supplementary Figure. 15. Normalized photovoltaic parameters of $EDAI_2$ device as a function of time and temperature, where the shaded areas refer to error bars derived from the standard deviation of respective photovoltaic parameters of three pixels subjected to rapid thermal cycling. a) Normalized *PCE* and temperature *versus* time, b) normalized J_{SC} and temperature *versus* time, c) normalized V_{OC} and temperature *versus* time, d) normalized *FF* and temperature *versus* time, and e) normalized hysteresis index and temperature *versus* time. Overall, the PCE of $EDAI_2$ device decreases to 46% of its original PCE after 1 h of thermal cycling, originating from the combined degradation of J_{SC} (6%), V_{OC} (15%), and FF (36%).

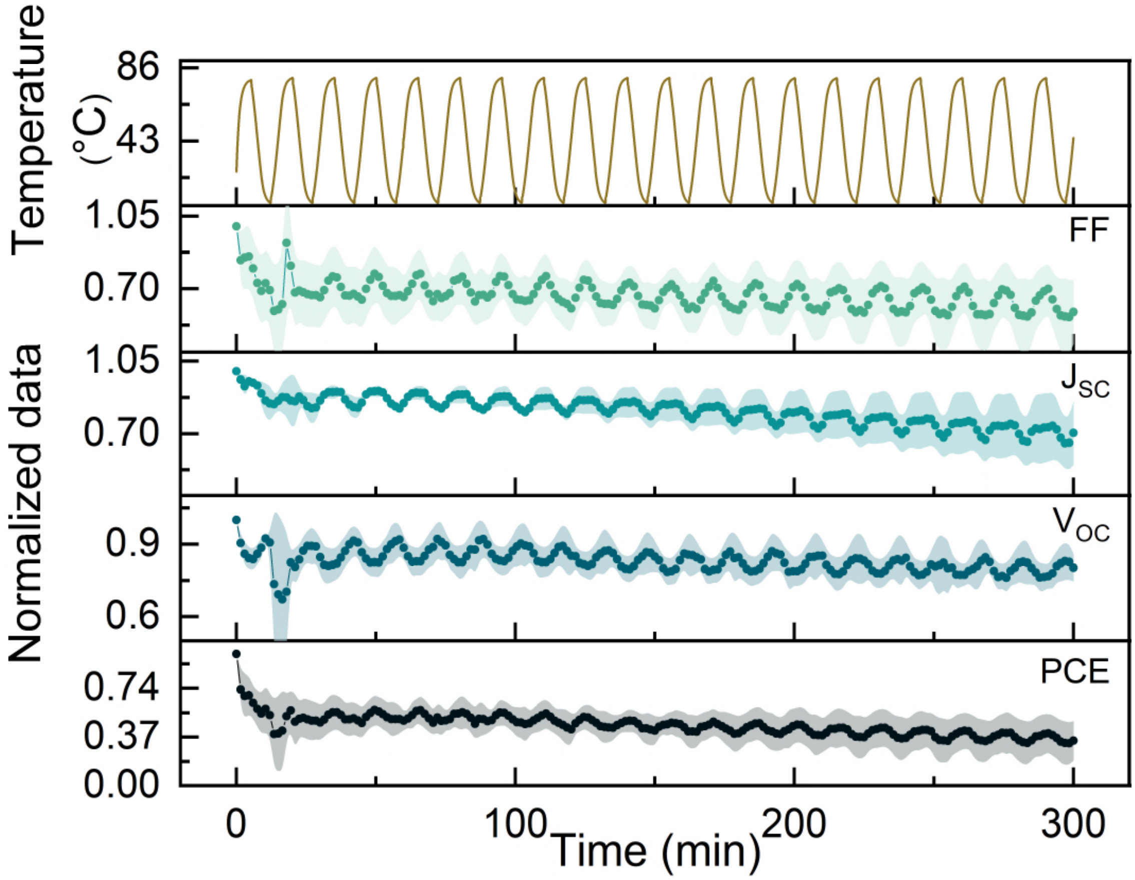

Supplementary Figure. 16. Normalized photovoltaic parameters of the control device as a function of time, from bottom to top: *PCE*, V_{OC}, J_{SC}, *FF*, and temperature, where the shaded areas refer to error bars derived from the standard deviation of respective photovoltaic parameters of three pixels subjected to rapid thermal cycling over a longer time.

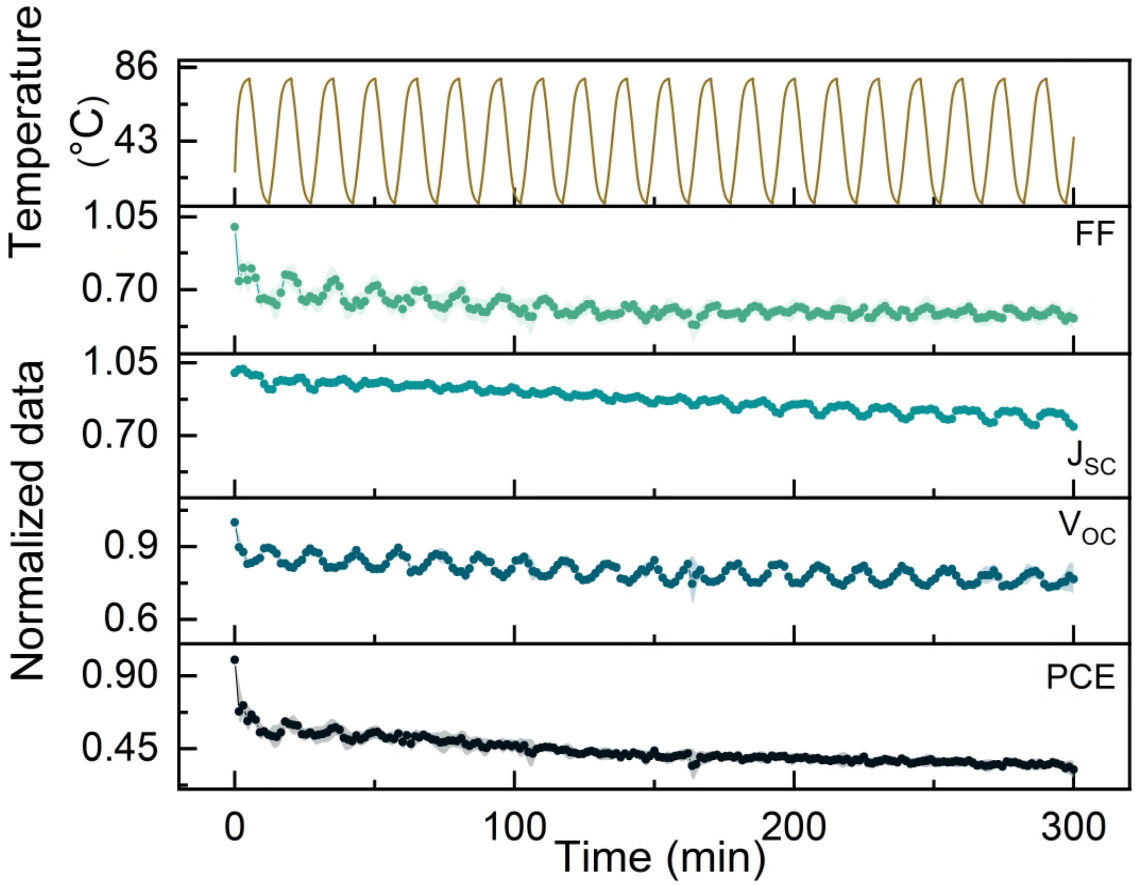

Supplementary Figure. 17. Normalized photovoltaic parameters of the $EDAI_2$ device as a function of time, from bottom to top: *PCE*, V_{OC}, J_{SC}, *FF*, and temperature, where the shaded areas refer to error bars derived from the standard deviation of respective photovoltaic parameters of three pixels subjected to rapid thermal cycling over a longer time. The PCE decreases to 32% of its original value, which is attributed to the 23% V_{OC} degradation, 26% J_{SC} degradation, and 46% degradation of *FF*.

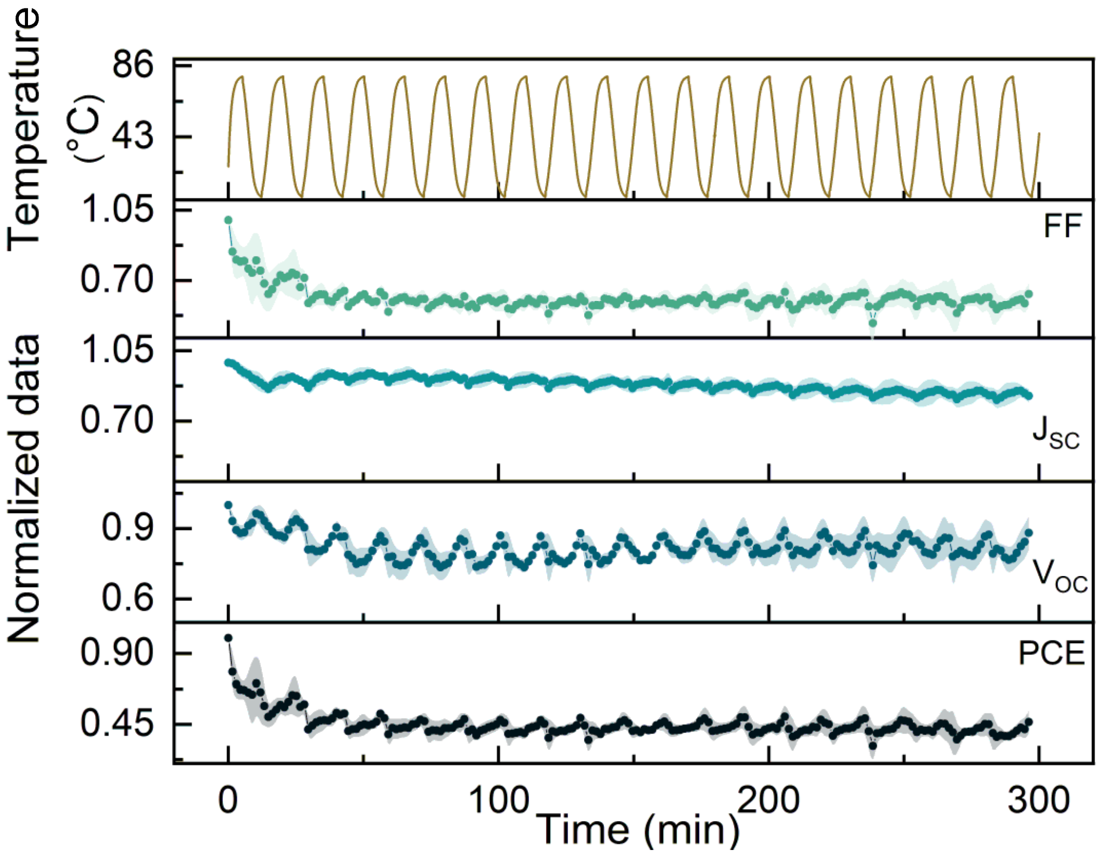

Supplementary Figure. 18. Normalized photovoltaic parameters of the DP device as a function of time, from bottom to top: *PCE*, V_{OC}, J_{SC}, *FF*, and temperature, where the shaded areas refer to error bars subjected to the standard deviation of respective photovoltaic parameters of three pixels operated under rapid thermal cycling over a longer time.

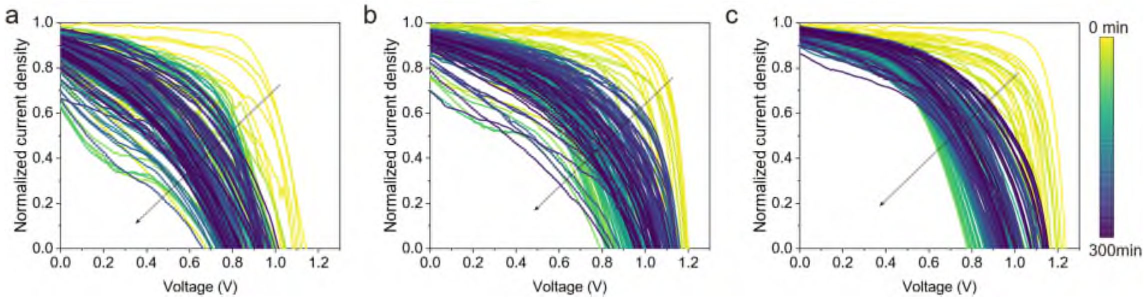

Supplementary Figure. 19. Normalized *J-V* curves as a function of time of the selected a) control device, b) $EDAI_2$ device, and c) DP device.

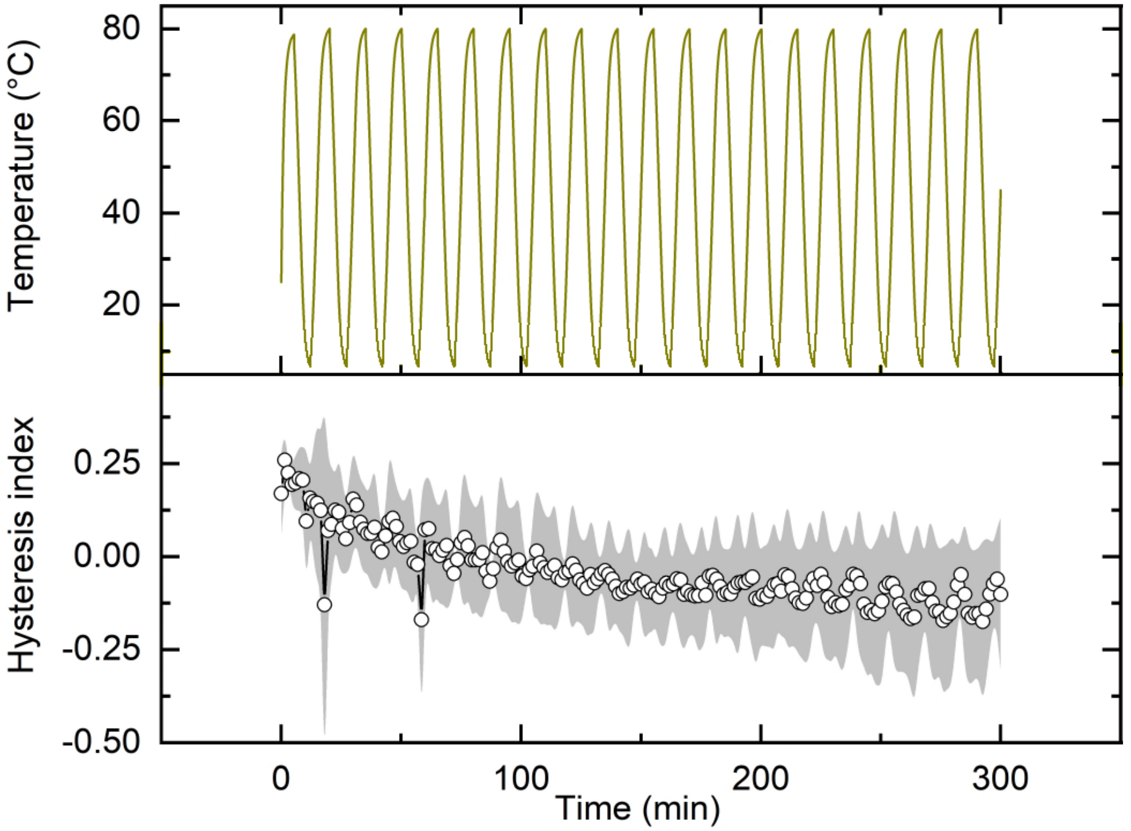

Supplementary Figure. 20. Time-resolved hysteresis index of the control devices under rapid thermal cycling for 300 min, where the shaded area indicates the error bars derived from the standard deviation of the hysteresis index across three devices.

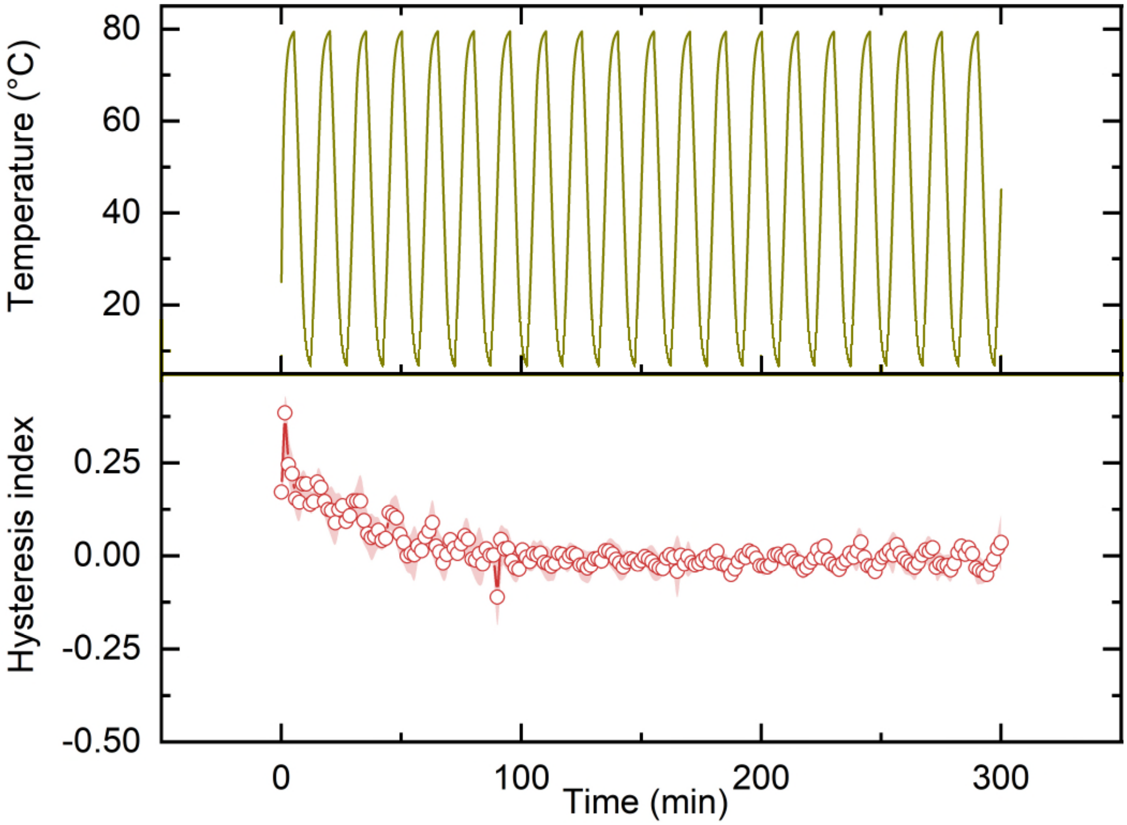

Supplementary Figure. 21. Time-resolved hysteresis index of the $EDAI_2$ devices under rapid thermal cycling for 300 min, where the shaded area indicates the error bars derived from the standard deviation of the hysteresis index across three devices.

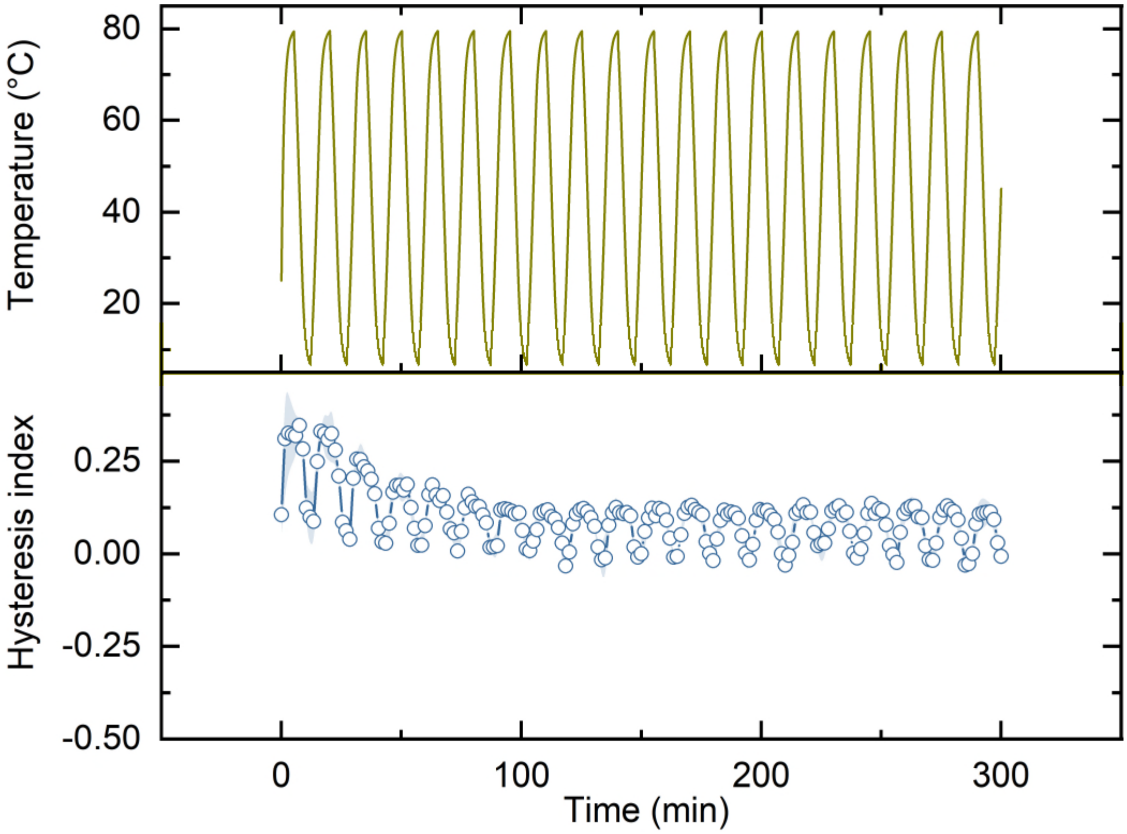

Supplementary Figure. 22. Time-resolved hysteresis index of the DP devices under rapid thermal cycling for 300 min, where the shaded area indicates the error bars derived from the standard deviation of the hysteresis index across three devices.

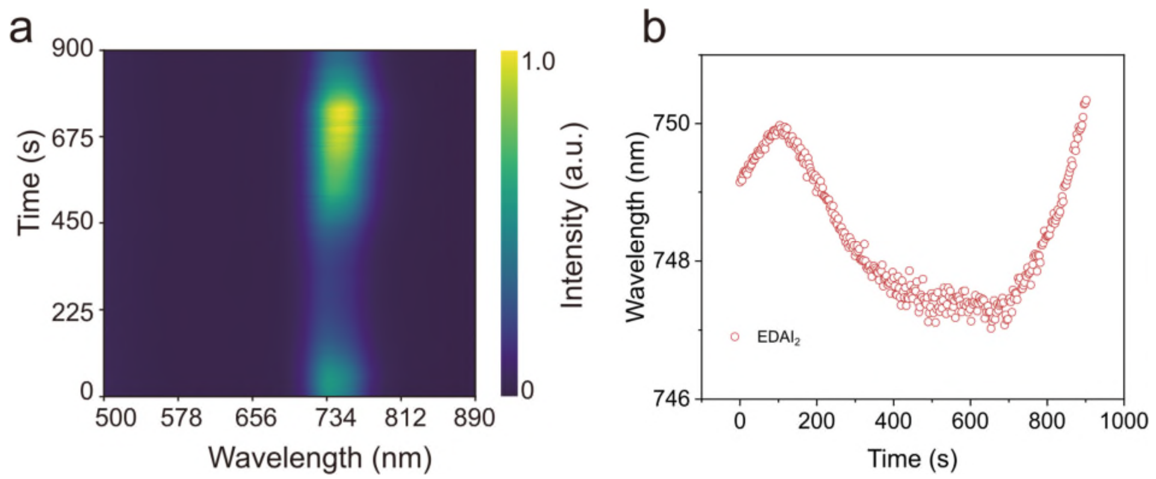

Supplementary Figure. 23. a) *In situ* photoluminescence evolution of $EDAI_2$ perovskite thin films as a function of time during thermal cycling, excited with a 450 nm laser. b) Peak position as a function of time, showing the minor change after $EDAI_2$ passivation.

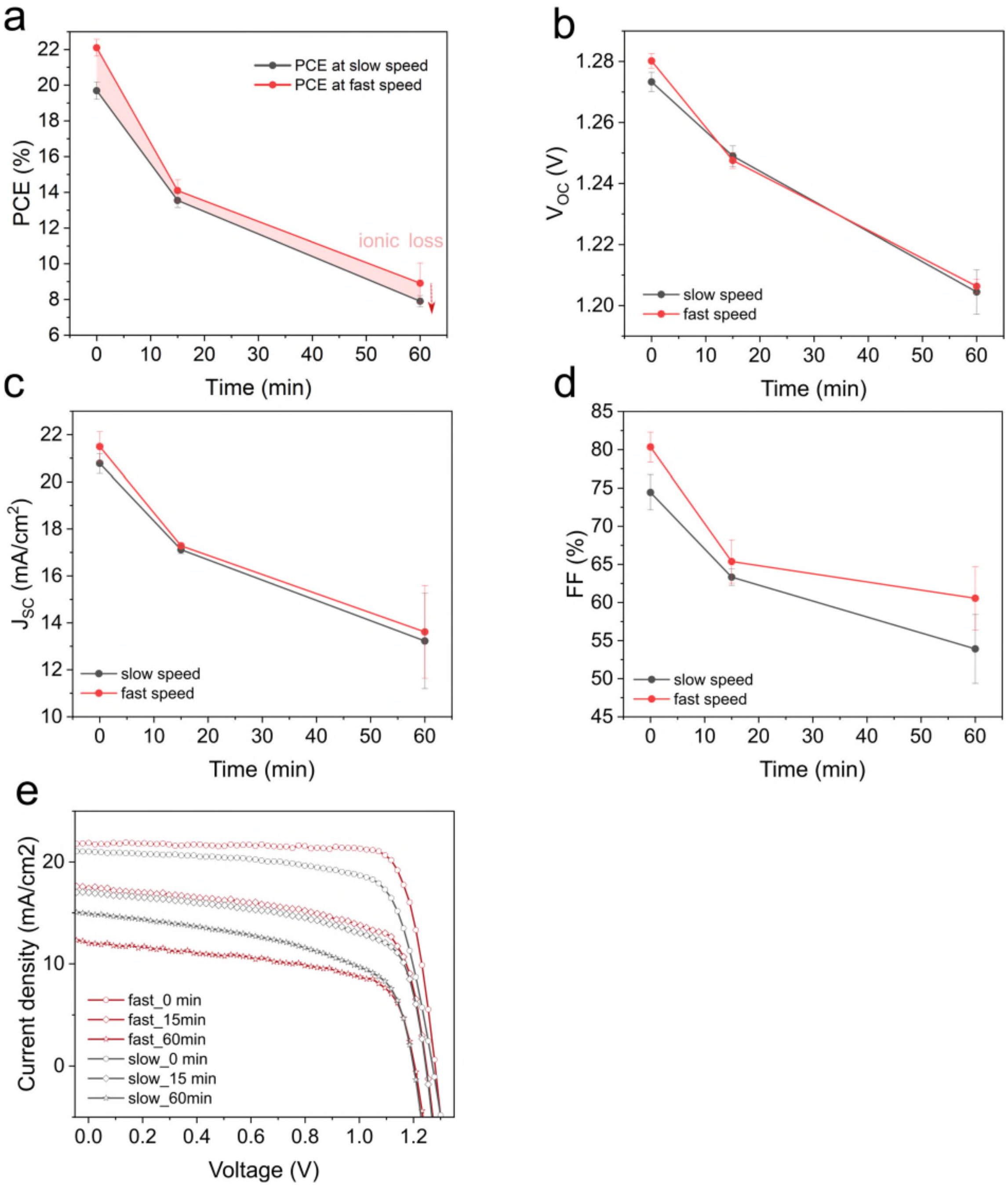

Supplementary Figure. 24. a) The absolute PCE at fast and slow scan speeds versus aging time under rapid solar-thermal cycling conditions. b) V_{OC} at fast and slow scan speeds versus aging time. c) J_{SC} at fast and slow scan speeds versus aging time. d) FF at fast and slow scan speeds versus aging time, and e) Exemplary *JV* curves measured at fast and slow scan speeds at different aging times.

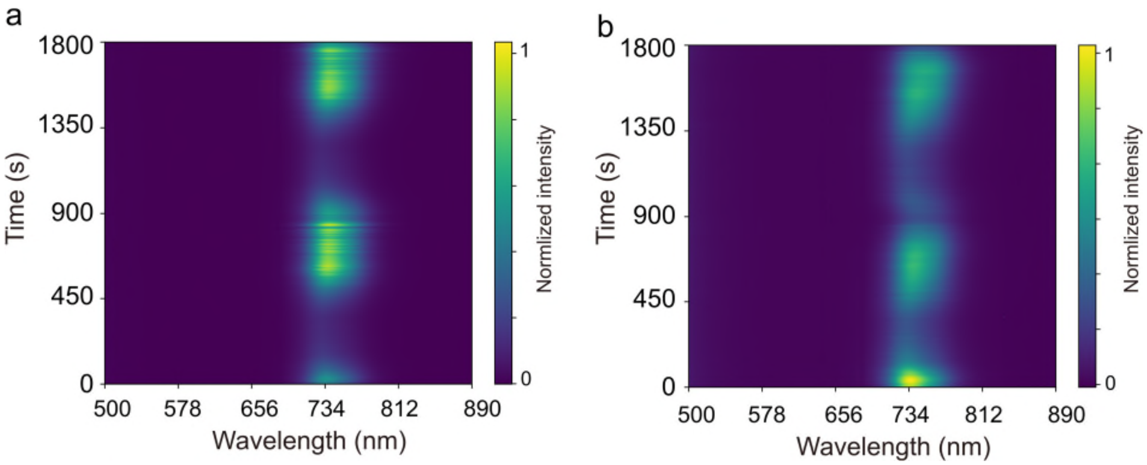

Supplementary Figure. 25. *In situ* photoluminescence evolution as a function of time over thermal cycles, excited with a 450 nm laser for a) control perovskite thin film and b) DP perovskite thin film.

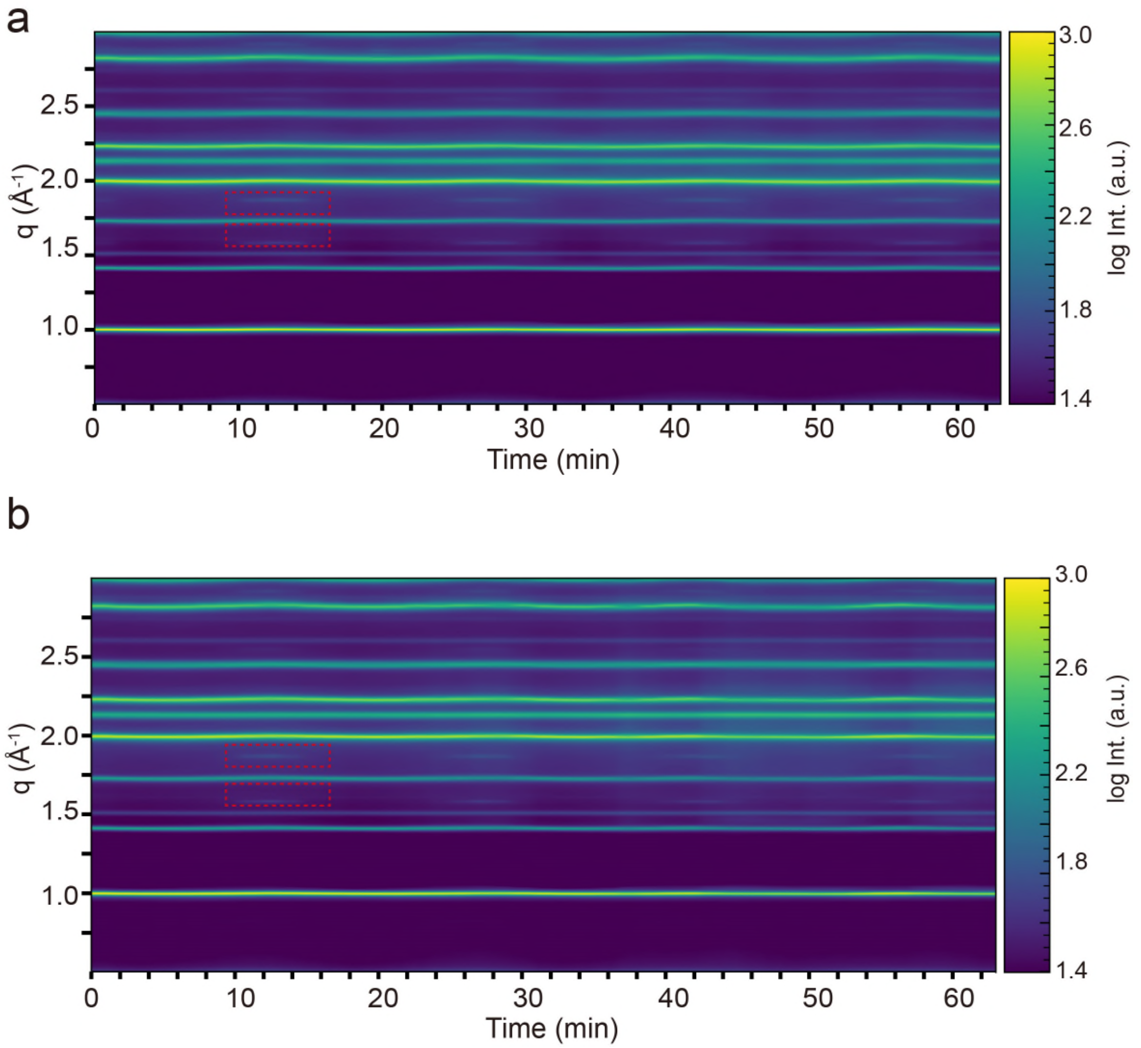

Supplementary Figure. 26. Time-resolved synchrotron-based GIWAXS under light illumination and thermal cycling conditions. a) control device and b) $EDAI_2$ device, showcasing the peak oscillations which align well with the temperature evolution. The red box indicates the reversible phase transition.

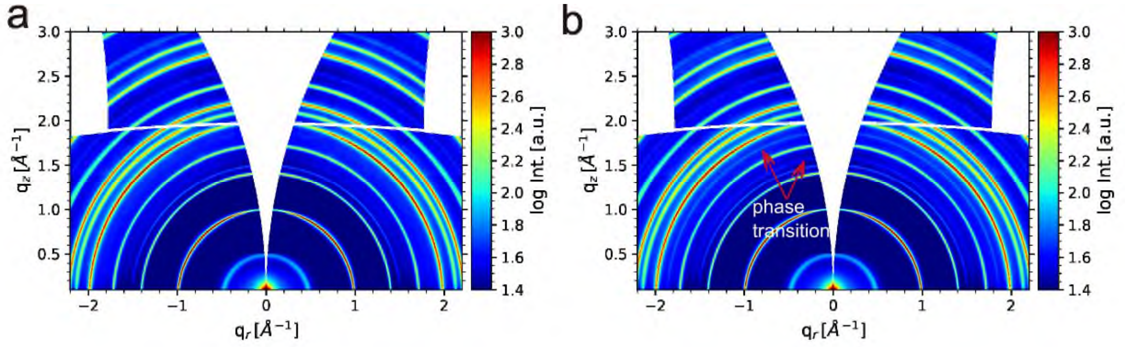

Supplementary Figure. 27. Selected reshaped 2D GIWAXS data of the control perovskite solar cells at a) 25 ℃ and b) at 5 ℃ within one thermal cycle, where the red arrows indicate the phase transition.

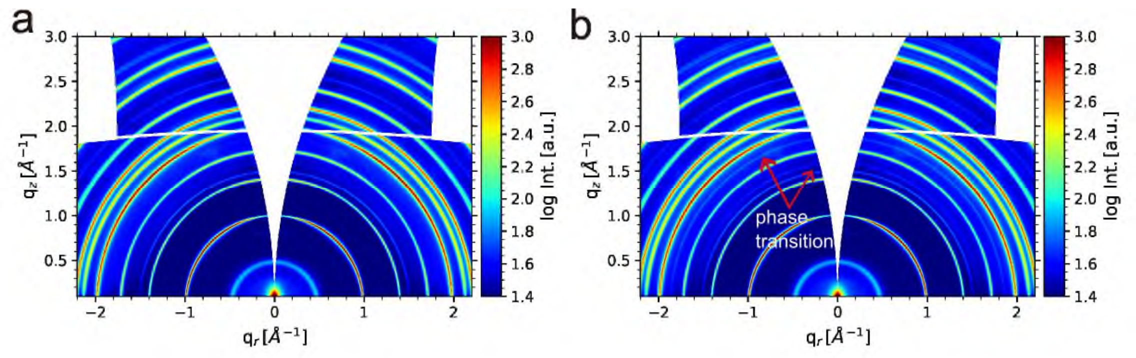

Supplementary Figure. 28. Selected reshaped 2D GIWAXS data of the $EDAI_2$ perovskite solar cells at a) 25 °C and b) at 5 °C within one thermal cycle, where the red arrows indicate the phase transition.

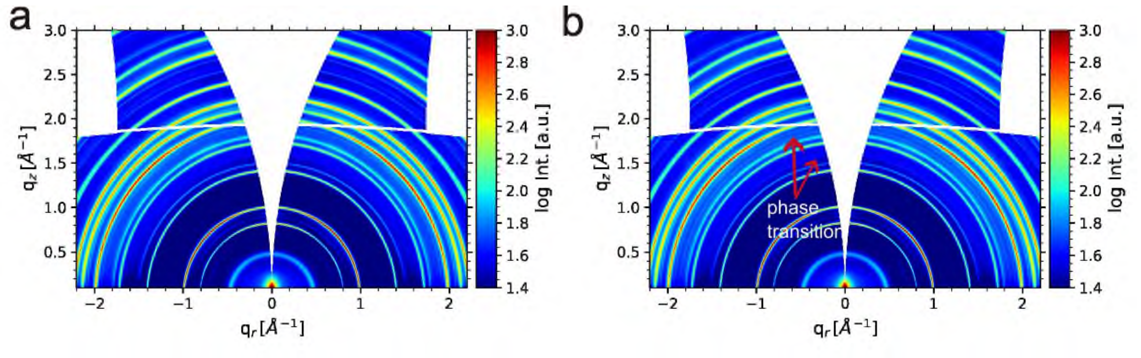

Figureure S29. Selected reshaped 2D GIWAXS data of data DP perovskite solar cells at a) 25 ℃ and b) at 5 ℃ within one thermal cycle, where the red arrows indicate the phase transition.

Supplementary Table 1. State-of-the-art perovskite solar cells with their thermal coefficients extracted from literature.

	**Perovskite composition**	**Perovskite configuration**	**Type**	**Temperature range (℃)**	**PCE at 25℃ (%)**	**Thermal coefficients**	**Ref**
**1**	$Cs_{0.05}(FA_{0.83}MA_{0.17})Pb_{1.1}(I_{0.83}Br_{0.17})_3$	ITO/MeO-2PACz/perovskite/C_{60}/SnO_2/Cu	SJ	25-85 ℃	18.3%	-0.17 $\%_{rel}$ K^{-1}	[3]
**2**	$FA_{0.79}MA_{0.16}Cs_{0.05}Pb(I_{0.83}Br_{0.17})_3$	ITO/PTAA/perovskite/LiF/C_{60}/BCP/Ag	SJ	-20-80 ℃	average ~17%	−0.36 rel %/°C	[4]
**3**	$FA_{0.75}Cs_{0.22}MA_{0.03}Pb(I_{0.82}Br_{0.15}Cl_{0.03})_3$	ITO/PTAA/perovskite/LiF/C_{60}/BCP/Ag	SJ	-20-80 ℃	average ~17%	-0.11 rel %/°C	[4]
**4**	N.A.	Perovskite/silicon tandem	TSCs	25-75 ℃	25%	−0.26 % K^{-1}	[5]
**5**	$MAPbI_3$	ITO/PTAA/perovskite/C_{60}/BCP/metal electrode	SJ	25-85 ℃	16.4%	−0.13 %/°C	[6]
**6**	$Cs_{0.10}FA_{0.90}Pb(I_{0.83}Br_{0.17})_3$	FTO/TiO_2/perovskite/Spiro-OMeTAD/Au	SJ	0-50 ℃	20%	N.A. non-monotonous	[7]

Supplementary References:

1. Tennyson EM, Doherty TA, Stranks SD. Heterogeneity at multiple length scales in halide perovskite semiconductors. *Nature Reviews Materials* **4**, 573-587 (2019).

2. Yoon SJ, Kuno M, Kamat PV. Shift happens. How halide ion defects influence photoinduced segregation in mixed halide perovskites. *ACS Energy Letters* **2**, 1507-1514 (2017).

3. Jošt M, *et al.* Perovskite solar cells go outdoors: field testing and temperature effects on energy yield. *Advanced energy materials* **10**, 2000454 (2020).

4. Moot T, *et al.* Temperature coefficients of perovskite photovoltaics for energy yield calculations. *ACS Energy Letters* **6**, 2038-2047 (2021).

5. Aydin E, *et al.* Interplay between temperature and bandgap energies on the outdoor performance of perovskite/silicon tandem solar cells. *Nature Energy* **5**, 851-859 (2020).

6. Deng Y, Van Brackle CH, Dai X, Zhao J, Chen B, Huang J. Tailoring solvent coordination for high-speed, room-temperature blading of perovskite photovoltaic films. *Science advances* **5**, eaax7537 (2019).

7. Tress W, *et al.* Performance of perovskite solar cells under simulated temperature-illumination real-world operating conditions. *Nature energy* **4**, 568-574 (2019).
