# Supplementary material for: Insights into the operational stability of wide-bandgap perovskite and tandem solar cells under rapid thermal cycling
Source: Nat Commun. 2026 Jan 14;17:596. doi: 10.1038/s41467-025-68219-w (PMC12808105; doi:10.1038/s41467-025-68219-w)
Supplement: Supplementary file 2 — Reporting Summary [file 41467_2025_68219_MOESM2_ESM.pdf]

nature portfolio

Corresponding author(s): Renjun Guo and Peter Müller-Buschbaum

Solar Cells Reporting Summary

Nature Portfolio wishes to improve the reproducibility of the work that we publish. This form is intended for publication with all accepted papers reporting the characterization of photovoltaic devices and provides structure for consistency and transparency in reporting. Some list items might not apply to an individual manuscript, but all fields must be completed for clarity.

For further information on Nature Research policies, including our data availability policy, see Authors & Referees.

• Experimental design

Please check the following details are reported in the manuscript, and provide a brief description or explanation where applicable.

1. Dimensions

Item	Yes	No	Description
Area of the tested solar cells	[x]	[ ]	Aperature areas used in the study are 0.05 cm2 (Fig. 1), 0.079 cm2 (Fig.2), and 1 cm2 (Fig.5) / *Explain why this information is not reported/not relevant.*
Method used to determine the device area	[x]	[ ]	Defined by the shadow mask / *Explain why this information is not reported/not relevant.*

2. Current-voltage characterization

Item	Yes	No	Description
Current density-voltage (J-V) plots in both forward and backward direction	[ ]	[x]	*State where this information can be found in the text.* / Negligible hysteresis
Voltage scan conditions	[x]	[ ]	The scan rate was appriximately 0.1-0.3 V/s for both reverse and forward scans / *Explain why this information is not reported/not relevant.*
Test environment	[x]	[ ]	Devices are measured in N2 without encapsulation / *Explain why this information is not reported/not relevant.*
Protocol for preconditioning of the device before its characterization	[ ]	[x]	*Provide a description of the protocol.* / No preconditioning protocol was uesd
Stability of the J-V characteristic	[x]	[ ]	Fig.1d and Fig.S9 in the manuscript / *Explain why this information is not reported/not relevant.*

3. Hysteresis or any other unusual behaviour

Item	Yes	No	Description
Description of the unusual behaviour observed during the characterization	[x]	[ ]	*Provide a description of hysteresis or any other unusual behaviour observed during the characterization.* / Fig.2b-e and the corresponding description in the manuscript
Related experimental data	[x]	[ ]	Fig.2b-e and the corresponding description in the manuscript / *Explain why this information is not reported/not relevant.*

4. Efficiency

Item	Yes	No	Description
External quantum efficiency (EQE) or incident photons to current efficiency (IPCE)	[x]	[ ]	Fig. 1c and Fig. S7 / *Explain why this information is not reported/not relevant.*
A comparison between the integrated response under the standard reference spectrum and the response measure under the simulator	[x]	[ ]	The integrated Jsc from the standard AM 1.5G spectrum matches the Jsc from the J-V measurmement within 5% / *Explain why this information is not reported/not relevant.*

For tandem solar cells, the bias illumination and bias voltage used for each subcell

- [x] Yes — *Provide a description of the measurement conditions.*
- [ ] No — *Explain why this information is not reported/not relevant.*

5. Calibration

Light source and reference cell or sensor used for the characterization

- [x] Yes — Detailed in Method section.
- [ ] No — *Explain why this information is not reported/not relevant.*

Confirmation that the reference cell was calibrated and certified

- [x] Yes — The light intensity was calibrated with a Si reference cell calibrated by Fraunhofer ISE.
- [ ] No — *Explain why this information is not reported/not relevant.*

Calculation of spectral mismatch between the reference cell and the devices under test

- [ ] Yes — Estimated mismatch factor is less than 1 and was therefore not applied.
- [x] No — *Explain why this information is not reported/not relevant.*

6. Mask/aperture

Size of the mask/aperture used during testing

- [x] Yes — Mask size 0.05, 0.079, 1 cm2
- [ ] No — *Explain why this information is not reported/not relevant.*

Variation of the measured short-circuit current density with the mask/aperture area

- [ ] Yes — *Report the difference in the short-circuit current density values measured with the mask and aperture area.*
- [x] No — All short-circult densities were measured with masks

7. Performance certification

Identity of the independent certification laboratory that confirmed the photovoltaic performance

- [ ] Yes — *Identify the independent certification laboratory.*
- [x] No — No certification was carried out since this is not the main focus of this manuscript

A copy of any certificate(s)

- [ ] Yes — *Certificate copies should be provided in the Supplementary information. Please state the supplementary item number.*
- [x] No — No certification.

8. Statistics

Number of solar cells tested

- [x] Yes — Fig.S6 in the SI
- [ ] No — *Explain why this information is not reported/not relevant.*

Statistical analysis of the device performance

- [x] Yes — Fig.S6in the SI
- [ ] No — *Explain why this information is not reported/not relevant.*

9. Long-term stability analysis

Type of analysis, bias conditions and environmental conditions

- [x] Yes — J-V measurements; AM 1.5G illumination, N2 atmosphere, thermal cycling (Details in the manuscript)
- [ ] No — *Explain why this information is not reported/not relevant.*
